# Radiation Therapy after Radical Prostatectomy: Implications for Clinicians

**DOI:** 10.3389/fonc.2016.00117

**Published:** 2016-05-09

**Authors:** Fernanda G. Herrera, Dominik R. Berthold

**Affiliations:** ^1^Radiation Oncology Services, Department of Oncology, Lausanne University Hospital, Lausanne, Switzerland; ^2^Medical Oncology Services, Department of Oncology, Lausanne University Hospital, Lausanne, Switzerland

**Keywords:** adjuvant radiotherapy, prostate cancer, androgen deprivation

## Abstract

Depending on the pathological findings, up to 60% of prostate cancer patients who undergo radical prostatectomy (RP) will develop biochemical relapse and require further local treatment. Radiotherapy (RT) immediately after RP may potentially eradicate any residual localized microscopic disease in the prostate bed, and it is associated with improved biochemical, clinical progression-free survival, and overall survival in patients with high-risk pathological features according to published randomized trials. Offering immediate adjuvant RT to all men with high-risk pathological factors we are over-treating around 50% of patients who would anyway be cancer-free, exposing them to unnecessary toxicity and adding costs to the health-care system. The current dilemma is, thus, whether to deliver adjuvant immediate RT solely on the basis of high-risk pathology, but in the absence of measurable prostate-specific antigen, or whether early salvage radiotherapy would yield equivalent outcomes. Randomized trials are ongoing to definitely answer this question. Retrospective analyses suggest that there is a dose–response favoring doses >70 Gy to the prostate bed. The evidence regarding the role of androgen deprivation therapy is emerging, and ongoing randomized trials are underway.

## Introduction

Prostate cancer is the most frequently diagnosed non-skin cancer in the western of world (1).

For such a frequent cancer, surprisingly, little certainties exist around its management.

After radical prostatectomy (RP), patients with high-risk pathological features, such as extracapsular prostatic extension (ECE), positive margins, seminal vesicle involvement (SVI), high Gleason score, and prostate-specific antigen (PSA) have a 40–70% risk of developing biochemical failure at some point in the future (2). Approximately, two-thirds of men with biochemical relapse will develop metastatic disease if left untreated (3).

Radiotherapy to the prostate loge has been used in both the adjuvant (ART) and the salvage (SRT) setting. Which of the two strategies is better remains an area of controversy despite the fact that there are three phase III randomized controlled trials that showed an improvement in biochemical progression-free survival (BPFS) when ART is administered as compared with RP alone (4–6). At the present time, there are no published randomized trials, and we dispose only of retrospective data for the use of SRT, making a direct comparison between ART and SRT flawed. While several trials comparing ART vs. SRT are on going, in this article, we summarize the available evidence on ART vs. SRT.

## Adjuvant Radiation Therapy Trials

Three randomized controlled trials are summarized in Table 1 showing the benefit of ART over RP alone. In these trials, ART was usually administered to the prostate loge within 4 months after RP (4–6). Two of the three randomized trials included patients with detectable PSA and therefore some patients actually received SRT, indirectly supporting its benefits over watchful waiting (4–6). With the introduction of ultrasensitive PSA, a new tool to detect low-volume disease became available, and therefore nowadays the term “adjuvant therapy” is used when the PSA is very low or undetectable (≤0.1 ng/ml) immediately or within 4 months of RP. This highlights the temporal variation in practice patterns and limits the generalizability of the results of the randomized trials to the contemporary population of prostate cancer patients. Nevertheless, these important studies provided evidence to support the use of postoperative RT in men with adverse pathologic features (ECE, SVI, or positive surgical margins). The question of whether all patients with the aforementioned adverse features should undergo immediate ART vs. initial observation with more selective – but early – SRT in the event of biochemical failure (using pre-defined PSA thresholds) remains a subject of controversy.

**Table 1:** **Randomized controlled trials comparing adjuvant postoperative radiotherapy vs. observation**.

Reference	*N*	Inclusion criteria	Dose (Gy)	Follow-up median (years)	10-year BPFS ART vs. NFT	10-year OS ART vs. NFT	10-year toxicity rate (%) ART vs. NFT
Thompson et al. (5)	425	pT3 cN0/pN0 R0/R1	60–64	12.7	52 vs. 26% *p* < 0.001	74 vs. 66% *p* = 0.023	GI, G3 = 3.3 vs. 0 GU, G3 17.8 vs. 9.5
Bolla et al. (4)	1005	pT2–3 pN0 R0/R1	60	10.6	60 vs. 41% *p* < 0.0001	77 vs. 81% *p* = 0.2	GU > G2 = 21.3 vs. 13.5 (*p* = 0.003) GI > G2 = 2.5 vs. 1.9 (*p* = 0.47)
Wiegel et al. (6)	388 (307)	pT3 pN0 R0/R1 PSA 0	60	9.3	56 vs. 35% *p* < 0.0001	84 vs. 86% *p* = 0.59	ART: GU, G3 = 1 patient, G2 = 2 patients, GI, G2 = 2 patients

*BPFS, Biochemical progression-free survival; OS, overall survival; ART, adjuvant radiation therapy; NFT, no further therapy; GU, genitourinary; GI, gastro-intestinal; G, grade*.

Certainly, men with prostate cancer will not necessarily die from the disease and even those who experience a biochemical failure will not necessarily become symptomatic from the disease (7).

Thus, the argument for postoperative radiotherapy (RT) is predicated on the assumption that some patients may have residual local disease of a potentially lethal phenotype after surgery and that the delivery of secondary local therapy may interrupt the natural history of disease and prevent progression to systemic disease. A basic question in this context is the extent to which this sequence of events – vs. the presence of occult metastases at surgery or the presence of a tumor that will never become symptomatic – characterizes the natural history of the disease.

### EORTC 22911

The European Organization for Research and Treatment of Cancer (EORTC) recruited 1005 patients between 1992 and 2001 to a randomized controlled trial (8). Patients with stage pT2–3, N0, M0 prostate cancer, and undetectable PSA defined as ≤0.4 ng/ml with at least one adverse prognostic factor: positive surgical margins, ECE and/or SVI were randomized to receive ART with 60 Gy in 6 weeks to the prostate bed or observation. After a median follow-up of 10.6 years, the intervention arm was significantly superior based on BPFS (74 vs. 53%; HR 0.49, 95% CI 0.41 to 0.59, *p* < 0.0001). The cumulative rate of loco-regional and any clinical failure was lower in the irradiated group (15 vs. 5%, *p* < 0.0001 and 19 vs. 9%, *p* < 0.0001, respectively). However, no significant benefit was observed in distant failures. Importantly, from the 265 patients in the observation arm who had biochemical progression 84% underwent an active treatment after progression (54.4% received pelvic radiotherapy, and 22.2% received androgen deprivation therapy-ADT). Salvage radiotherapy was administered to 23% of patients in the observation group. There was also a significant increase in late side effects of any type and any grade in the RT arm [10-year cumulative incidence 70.8% (66.6–75.0) vs. 59·7% (55.3–64.1); *p* = 0.001]. After 10 years of follow-up, improvements in clinical progression-free survival vanished, and overall survival (OS) was not improved (4).

### SWOG 8794

From 1988 to 1997, the South West Oncology Study Group (SWOG) 8794 trial randomized 430 men with pT3, pN0, M0, ECE, positive margins, and/or SVI prostate cancer to ART (60–64 Gy) or observation (5, 9). There was no restriction on PSA level at enrollment. The primary endpoint was metastasis-free survival. Secondary endpoints were PSA relapse, recurrence-free survival, OS, and postoperative complications. With a median follow-up of 12.7 years, the study was positive for metastases-free survival favoring the RT arm (43 vs. 54%, HR 0.71, 95% CI 0.54–0.94; *p* = 0.016). Also the OS was improved significantly with ART (41 vs. 52%, HR 0.72, 95% CI 0.55–0.96; *p* = 0.023).

Swanson et al. reported that the pattern of failure was local with 22% of patients having a clinical local failure in the observation arm compared to 8% in the ART arm. An additional 11 patients in the observation arm had local and distant failures compared to 1 patient in the treated arm (10). The time to initiation of hormonal therapy differed in both groups with 21% of patients in the observation group having received ADT within 5 years post biochemical relapse vs. 10% of patients in the ART group (HR 0.45; 95% CI 0.29–0.68, *p* < 0.001) (9).

It has, however, been argued that the survival benefit may be due to hazard considering that the trial was not powered to detect an OS advantage as well as the fact that such clinical benefit was not found in the European Study (11). The most significant problem with the SWOG 8794 trial was the lack of disclosure of cause of death. There were 110 men (52%) who died in the observational group vs. 88 (41%) in the radiation group, but because of the long follow-up period it was not possible for the authors to ascertain if these patients died from metastatic prostate cancer or from competing hazards, which jeopardizes the impact of ART on OS.

Another observation from this trial is that the pre-radiation PSA level immediately after RP was predictive of subsequent outcome. For instance, for patients with an undetectable PSA (≤0.2 ng/ml), the 5-year PSA failure rate was 77% (very similar to the EORTC 22911 trial, 74%). However, patients with a postprostatectomy PSA between 0.2 and ≤1 ng/ml had a PSA failure rate of 34% at 5 years: this last group had an 8% increase in the risk of metastases indicating thus that RT with these PSA values is less efficient in eradicating larger tumor deposits (10). On the contrary, patients in the observation arm had a significant delay in initiating SRT. The median PSA at which patients were referred for salvage radiation was 1–1.5 ng/ml. A better comparison would have been made if patients in the observation arm had been offered RT at the first PSA failure.

### ARO 96/02

The German study was the only real adjuvant study as the inclusion criteria permitted only patients with undetectable PSA. Those with persistently detectable PSA after surgery were declared as having progressive disease. The study recruited 307 patients from 1997 to 2004. Eligible patients had pT3 pN0 tumors with positive or negative margins. PSA failure was defined as two consecutive rises above undetectable. BPFS after 5 years was significantly improved with ART (72%, 95% CI 65–81%, vs. 54%, 95% CI 45–63%, HR = 0.53, 95% CI 0.37–0.79, *p* = 0.0015). Despite a 10-year follow-up period, the study did not show any clinical benefit.

Grade 2 genitourinary and gastrointestinal toxicity in the RT arm were 2 and 1.4%, respectively (6).

## Who Might Benefit from Immediate Postoperative Radiotherapy?

In summary, the 3 studies included together over 1100 patients and had a substantial follow-up assessment that permits some conclusions: ART compared with watch and see strategy reduced by about 20% the risk of PSA relapse.

Van der Kwast et al. reviewed about 50% of the pathology specimens in the EORTC 22911 trial, in particular with regard to positive surgical margins (12). After 5 years, immediate postoperative radiation was shown to prevent 191 events in 1000 patients with positive margins vs. 88 events in 1000 patients with negative margins. The hazard ratio for immediate radiation was 0.38 (95% CI 0.26–0.54) and 0.88 (95% CI 0.3–1.46) in the group with positive and negative margins, respectively. The finding of a significant association between margin status and adjuvant RT benefit was also reported in the subgroup analyses of ARO 96-02 (6). The data indicate that patients with a pT3 tumor but negative margins may potentially benefit less or not at all from immediate ART. Exploratory analyses suggested that postoperative irradiation might improve clinical progression-free survival in patients younger than 70 years old. Radiotherapy could have a detrimental effect in patients aged 70 years or older (13).

An important point to consider in the equation balance between ART and observation is that one-third of the patients in the wait and see arm of the randomized trials have received ADT within the first 5 years after biochemical progression. Therefore, the use of ART would lead to a diminished use of ADT. However, early initiation of ADT has not shown convincing benefit on OS (14, 15).

It is also important to note that the standard routine clinical practice has evolved since the publication of the three aforementioned randomized trials, and contemporary patients have access to ultra-sensitive PSA assays, consequently in modern clinical practice patients with an undetectable postoperative PSA level have a lower risk of relapse than those patients in the past and so less potential benefit from adjuvant immediate radiation. Ultra-sensitive PSA assays also lead to an earlier detection of biochemical relapse than clinical relapse, and this early detection may lead to an improvement in the efficacy of SRT as a therapeutic option. There is at the moment no consensus among clinicians on whether to use adjuvant or salvage radiotherapy, highlighting the need for offering these patients a randomized trial (16).

The potential for toxicity needs to be considered when counseling patients for postprostatectomy RT, and this information provides support for more selective use.

## Salvage Radiation Therapy

Salvage radiation therapy is supported by some clinicians based on the rationale that an elevated PSA in the postoperative setting or a delayed PSA rise is caused, at least in some patients, by the persistence of local disease. However, while the disease is localized to the surgical bed and curable with SRT, the presence of occult metastatic disease cannot be excluded. Certainly, adverse prognostic factors in the pathology specimen such as ECE, positive margins, and SVI, support the concept of a local residual tumor and thus the use of salvage treatment (17). The lower the PSA at the time of salvage therapy, the better the outcome. Investigators have tried to use PSA cut-off points ranging from ≥0.1 to 10 ng/ml (18–23). However, it should be noted that the relationship between pre-radiotherapy PSA and radiotherapy outcome is a continuum. In general, PSA recurrence rates have been reported to be higher when the PSA is ≥0.2 ng/ml (20, 21, 24). Other factors should also be considered such as the PSA doubling time as well as time to biochemical failure (25).

There have been multiple retrospective studies, which have looked at the clinical question of how adjuvant or salvage radiation affects local control, BPFS, and OS. Significant improvements in local control and BPFS have been observed in patients treated in the adjuvant or early adjuvant setting compared to those treated with late salvage therapy. The inherent caveat when comparing ARC vs. SRT in retrospective studies is that the patient receiving salvage treatments have in most of the cases confirmed recurrent disease, consequently the outcome of salvage radiation will always seem worse than ART (11). The 5-year BPFS rates were approximately 59–80% after ART (19–21) and 26–66% after salvage radiotherapy (22, 26). In some series, patients who had an undetectable PSA after SRT had a 75% chance to have an undetectable PSA at 3.5 years (6).

Table 2 summarizes the retrospective studies on SRT. It is clear from these retrospective studies that oncological outcomes are better when SRT is initiated at the lowest PSA values. Several studies showed that the biochemical relapse free survival can be >75% with pre-radiotherapy PSA of <0.5 ng/ml (24, 27).

**Table 2:** **Selected series of salvage radiotherapy for PSA relapse after radical prostatectomy**.

Reference	*N*	Comparison	PSA pre-RT (ng/ml)	ADT (%)	Median follow-up (months)	BPFS (%)	Important prognositc factors	RT technique/dose (Gy)	Grade 3 toxicity (%)
Pfister (27)	737	Early salvage	<0.5	6.7	51	71	PSA pre RT <0.2	2D/3D/IMRT	0.6–1.3
Trock (23)	160	SRT with PSA >0.2–22	Median 0.7	12	72	89		2D/3D/66.5	
Swanson (26)	92	ART (*n* = 36) with postoperative PSA <0.4 vs. SRT (*n* = 56)	Median 1.5	0	146.4	35 vs. 25	GS >8 PSA >0.5	2D/3D/60–70	NR
Trabulsi (22)	449	ART <12 months from surgery (*n* = 211) SRT >12 months from surgery (*n* = 238)	<2	0	94	75 vs. 66	GS >8	Use of SRT	2D/3D/64	NR
Fossati (20)	955	Early salvage	<0.5	0	57	82	PSA >0.2, >pT3, GS >7	2D/3D/66.6	NR
Cremers (19)	197	SRT (>6 months after RP)	45.7% with PSA <10 and 53.8% with PSA > 10	0	40	59	GS >7, ECE, PSA >1ng/ml	3D/66	GU = 6 GI = 0.6
Jereczek-Fossa (21)	431	ART <6 months after RP (*n* = 258) SRT >6 months after RT (*n* = 173)	ART 0–4 SRT 0.1–13.7	100	32	81 vs. 60.5	PSA >0.2 GS >6 Age <65	70	GI = 0.7 GU = 1.9
Briganti (24)	390	PSA <0.3 vs. PSA >0.3 to <0.5	58	0	40.6	81.8	stage, GS, and positive SM	3D/66.2	NR
Siegmann (28)	301	SRT (median time to RT 23 months) In 151 patients, SRT commenced at PSA ≤0.28 ng/ml, in 150 at >0.28	0.28	0	30	78 vs. 61% for a PSA ≤ or >0.28 ng/ml	pT3b, positive SM, pre-SRT PSA, PSA doubling time	3D/68.4	GU = 1.3
Stephenson (2)	1540	Nomogram for disease progression after SRT	<0.5 to ≥0.5	0	53	PSA <0.5 = 48, PSA >0.51–1.00 = 40, PSA 1.01–1.50 = 28, PSA >1.50 = 18	GS, PSA doubling time, SM, ADT	64.8	NR

*GS, Gleason score; PSA, prostate-specific antigen; RT, radiotherapy; ADT, androgen deprivation therapy; BPFS, biochemical progression-free survival; ART, adjuvant radiation therapy; SRT, salvage radiation therapy; RP, radical prostatectomy; SM, surgical margins; GU, genitourinary; GI, gastro-intestinal; NR, not-reported; 2D, two dimensional radiotherapy; 3D, three-dimensional conformal radiotherapy*.

A recent report by Pfister et al. analyzed 10 retrospective reports on patients with early salvage radiotherapy (ESRT) (27). The term ESRT refers to patients with undetectable PSA after prostatectomy who have subsequent PSA rise ≤0.5 ng/ml. Significantly, increased cancer control rates have been reported with ESRT compared to late SRT. The mean 5-year biochemical relapse-free survival was 71% in a polled analysis of 886 patients treated with ESRT. However, no data on clinically outcomes such as metastasis-free survival or OS were available. Siegmann et al. (28) reported a BPFS of 83% at 2 years for patients with PSA ≤0.2 ng/ml at the time of SRT compared to 61% for those with a PSA of ≥0.28 to ≤1 ng/ml, pointing out that further reducing the PSA cut-off point may increase biochemical outcomes.

The nomograms introduced by Stephenson et al. may help in the decision-making process (2). This nomogram was created from a pooled database of 1818 patients with a median follow-up of 53 months after RT. Pre-surgical prognostic factors such as PSA, Gleason score, SVI, ECE, surgical margins, lymph node status, PSA at SRT, PSA doubling-time, time-to-recurrence, time from recurrence to radiation, radiation dose, and the use of ADT are considered to allow individualized risk stratification. Briganti et al. (24) restricted the nomogram to patients with PSA <0.5 ng/ml and validated it with 200 bootstrap resamples demonstrating a good discrimination in outcome with a *c*-index of 0.74. By incorporating genomic tests into nomogram models, Den et al. (29) analyzed 188 patients with pT3 or positive margin prostate cancer looking at 22 pre-specified gene-signatures and reported an improvement in the Stephenson nomogram from a *c*-index of 0.70–0.80 for BPFS as well as distant metastases. Novel gene signatures describing the biology of prostate cancer progression have recently being summarized in a comprehensive review (30). Table 3 describes biomarker studies in the postoperative setting (31–34).

**Table 3:** **Selected biomarkers tested in the postoperative setting**.

Reference	*N*	Biomarker	Assay	Adverse prognostic factor for:
Den et al. (29)	188 (T3, margins positive)	22 genes	Tumor-derived RNA	Score ≥0.4, 6 vs. 23% probability of metastases for adjuvant vs. salvage RT
Parker et al. (33)	147	Ki-67	IHC	BR after SRT
Cuzick et al. (31)	366	31 cell cycle progression genes	Tumor-derived RNA	BR after radical prostatectomy defined as PSA >0.3
Wu et al. (34)	270	32 genes	Tumor derived real-time PCR	BR after RP >20% risk if index score >3
Erho et al. (32)	546	22 genes of cell proliferation and mobility	Tumor-derived RNA	BR after RP and metastatic progression

*BR, Biochemical relapse; SRT, salvage radiation therapy; RP, radical prostatectomy; IHC, immunohistochemistry; RNA, ribonucleic acid; PCR, polymerase chain reaction*.

Certainly, when analyzing this data, one must consider that not all the patients treated with a PSA under 0.2 ng/ml will benefit clinically from SRT. The natural history of men with PSA relapses after RP is long: in the series described by Freedland et al., the median survival has not been reached after 16 years of follow-up. Patients at risk of prostate cancer death had shorter time to relapse, shorter PSA doubling times, and higher Gleason scores (7).

So far, only a few retrospective analyses have data on clinical significant endpoints. In the analysis by Boorjian et al., ART and SRT were independent predictors for biochemical and local control. In addition, SRT decreased the rate of systemic failures (35). Jereczek-Fossa et al. reported on 431 patients treated between 1996 and 2006: 258 men received immediate RT for a rising PSA between 0.1 and 4 ng/ml vs. 173 men who received SRT >6 months after surgery for a rising PSA that was between 0.1 and 13.7 ng/ml. Interestingly, in this study >78% of patients had biopsy-confirmed prostate relapse at the time of SRT, and 10 patients had palpable disease in the prostate bed. After a median follow-up time of 48 months, failure-free survival including BPFS and clinical failure was significantly longer in the immediate RT group (79.8 vs. 60.5%, *p* < 0.0001). In multivariate analysis, pre-radiotherapy PSA ≥0.2 ng/ml (*p* < 0.001) correlated with worst clinical outcome highlighting the more advanced tumors included in the SRT group (21). Swanson et al. reported a series of 92 patients referred to SRT for a rising PSA level at the time of referral from 0.1 to 30.5 ng/ml (median 1.5 ng/ml). The median time from surgery to radiation was 2.1 years (range 0.3–7.4 years). After a median follow-up time of 12.2 years, the 5- and 10-year BPFS was 35 and 26%, respectively, and OS was 86 and 67%, respectively. The median biochemical-free survival after SRT was 2.3 years (26).

The benefits of SRT should always be balanced against the morbidity of the therapy. Many large retrospective series assessing oncological outcomes after SRT did not include long-term toxicity data. In a retrospective series of 742 patients who underwent ART or SRT, the incidence of acute toxicity grade 2 or more was 19% after ART and 17% after SRT. The incidence of grade 3 toxicity was 8 and 6%, respectively. No differences in grade 2 or more late toxicity were observed. However, there were slightly more grade 3 late toxicity events in the ART group (12.2 vs. 10%) (36).

## Salvage Radiotherapy and Androgen Deprivation Therapy

GETUG-AFU 16 was the first randomized trial comparing SRT vs. SRT and short ADT as salvage treatment for biochemical recurrent prostate cancer after radical prostatectomy and was presented in abstract form at the American Association of Clinical Oncology (ASCO) 2015 Annual Meeting. The trial randomized 743 patients most of them having high intermediate risk features (pT2ac: 54%, pT3ac: 46%, gleason >6: 76%, positive margins: 51%, seminal vesicles’ involvement 13%, and PSA doubling time at relapse was >6 months in 74%). The 5-year PFS was 62.1% (CI 95%: 57–67) vs. 79.6% (CI 95%: 75–84) for SRT and SRT + ADT, respectively (*p* < 0.0001). The 5-year OS was 94.8% for RT vs. 96.2% for SRT + ADT (*p* = 0.18). Cause of death was progressive disease in 2.1% of the patients on SRT arm vs. 0.8% in the SRT + ADT arm. Acute toxicities occurred more frequently in SRT + ADT arm (89 vs. 79%). This trial will require longer follow-up to see if the benefits observed in progression-free survival translate into the same OS benefit (37).

A recent phase I/II study evaluated 75 patients with PSA relapse after RP who were treated with SRT followed by 2-year ADT. Androgen ablation therapy started within 1 month after the completion of SRT. The study used a PSA rise above 0.2 ng/ml with two consecutive increases over a minimum of 3 months as the definition of PSA relapse post-therapy. All achieved initially complete PSA response (<0.2 ng/ml) with the protocol treatment. With the median follow-up of 6.4 years (range: 2–9.8) from SRT, the study reported that a relapse-free rate including the freedom from PSA relapse was 91.5% at 5 years and 78.6% at 7 years, and OS rate was 93.2% at both 5 and 7 years (17). Some retrospective data suggest that adding ADT to SRT increases patient’s BPFS outcome. Tiguert et al. published a 5-year BPFS rate of 50% for 81 patients treated with 3 months of neoadjuvant ADT followed by SRT (38). In another series of 115 patients, 45 patients received 3 months of ADT followed by SRT and 70 patients were treated with SRT alone. The 4-year BPFS was better for patients treated with neoadjuvant ADT (59 vs. 39%) (39). King compared treatment outcomes between SRT plus 4-month ADT (2-months before and 2-months during RT) and SRT alone in a retrospective study of 122 patients (40). A 5-year BPFS rate was better for those treated with SRT plus 4-month ADT than for those receiving SRT alone (57 vs. 31%). Taylor et al. (41) reported on 35 out of 71 patients treated with adjuvant ADT for a median duration of 24 months. After a median follow-up of 39 months, the 5-year BPFS rate was 81% for patients receiving adjuvant ADT, compared with 54% for those treated with SRT alone.

On the other hand, Trock et al. published a retrospective analysis of 635 men treated from 1982 to 2004 who received no SRT (*n* = 397) with a median PSA level of 9.6 ng/ml, SRT (*n* = 160) with a median PSA level of 8.3 ng/ml, and SRT combined with ADT (*n* = 78) who had a median PSA level of 7.7 ng/ml (23). The groups were otherwise not well balanced regarding pathological and clinical factors and patients who had SRT combined with ADT had a shorter time to recurrence, shorter PSA doubling time, and a higher PSA level at the time RT was initiated. The primary outcome was prostate cancer-specific survival defined from time to recurrence to death. SRT alone was associated with a significant threefold increase in prostate cancer-specific survival relative to those who received no further treatment (HR 0.34, 95% CI 0.17–0.69, *p* = 0.003). In this study, the addition of ADT was not associated with any additional increase in prostate cancer-specific survival. Notably, patients in the no-SRT group had a much higher prevalence of positive pelvic lymph nodes at recurrence.

## Radiological Assessment

It should also be noted that patients undergoing SRT should be correctly staged. However, conventional imaging investigations such as bone scan and computed tomography of the chest, abdomen, and pelvis have been very insensitive for patients with biochemical-relapsed prostate cancer after RP. Nevertheless, we perform these tests in our routine clinical practice because the detection of any distant metastasis obviates the need for local salvage treatment. Cher et al. reported in a series of 93 patients with PSA relapse that the probability of a positive bone scan was <5%, unless a PSA level was above 40 ng/ml (42).

Similarly, the sensitivity of abdominopelvic CT scans is limited when PSA levels are low. Okotie reported that when the PSA was <10 ng/ml, the probability of a positive CT scan was non-existent (43). However, the use of MRI has enabled clinicians to assess the prostate bed more accurately. Miralbell et al. showed that MRI was capable of documenting a recurrent or residual disease in the setting of PSA levels ranging from 0.05 to 13.3 ng/ml (median: 0.87), typically in the inferior and posterior region of the vesicourethral anastomosis (44).

The use of conventional positron emission tomography (PET) tracers such as ^18^F-fluorodeoxyglucose (FDG) is of no help in prostate cancer due to a low glycolysis rate and the renal excretion of the isotope into the bladder, enabling any local uptake.

In this context, recent studies showed that for patients with biochemical recurrence choline PET/CT may visualize the site of recurrence earlier and with higher accuracy than conventional imaging modalities. Rinnab et al. reported that ^11^C-choline PET/CT had a sensitivity of 89% and a specificity of 40% for patients with post-RP PSA levels <2.5 ng/ml (45). A higher PSA level, PSA velocity, and PSA doubling time are predictive factors for having a positive ^11^C-choline PET/CT (46). In a series of 21 patients with post-RP PSA relapse (median PSA: 1.98 ng/ml), ^11^C-choline PET/CT improved the detection of lymph node metastases that were subsequently confirmed by histological assessment in 19 of the 21 patients (90%) (47). On a nodal site-based analysis, it was estimated that the sensitivity, specificity, positive predictive value, negative predictive value, and accuracy of ^11^C-choline PET/CT was 64, 90, 86, 72, and 77%, respectively (48).

The information gained with PET/CT in this clinical setting has the potential to change disease management. In a recent clinical series reported by Alongi et al., 15 patients with biochemical recurrence after HIFU therapy and a median pre-RT PSA of 5.2 ng/ml (range: 2–64.2) underwent 11 C-choline PET/CTs, documenting intra-prostatic-only failure and allowing a better tailored salvaged treatment using volumetric modulated arc therapy (VMAT) (49).

Conversely, a recent study raised some doubt over the sensitivity of PET/CT in the clinical setting of low PSA levels. Vees et al. reported, in a series of 20 patients with post-RP PSA levels ≤1 ng/ml, that only 11 were found to have a positive PET/CT using either ^18^F-choline or ^11^C-acetate (50).

This highlights the fact that PET/CT often remains negative in early relapse situations when PSA levels are still very low (<1 ng/ml). Unfortunately, these levels are the “window” where ESRT will be most effective. Other tracers such as Ga-68-Prostate Specific Membrane Antigen (PSMA) are emerging in recent literature with preliminary promising results (51, 52).

## Radiation Dose, Technique, and the Effect of Dose Escalation

Traditionally, the three randomized trials for ART used 60–65 Gy typically with 3D simulation (4–6). In some cases, the treatment volumes were typically very generous being described as approximately 10 cm × 10 cm in the anterior–posterior fields with the inferior border at the ischial tuberosities. The lateral fields extended from the anterior aspect of the pubic symphysis and split the rectum posteriorly (8). In 3-Dimensional Conformal Radiation Therapy (3D-CRT), the target volume should include the bladder neck (pulled into the prostate bed), periprostatic tissues/clips, and the seminal vesicle bed (including any seminal vesicle remnants if present). Inferiorly, the vesicourethral anastomosis should be included. The anastomosis is the most frequent area of positive prostate biopsies (53, 54). By placing the inferior field edge at the top of the bulb of the penis and adding a margin for uncertainties, there should be adequate coverage. Laterally, the field should extend to about the medial aspect of each obturator internus muscle. Although the rectum is a landmark posteriorly, and its movement has been a matter of possible target missing, for this reason, a generous margin posteriorly is recommended in international guidelines (55). The superior margin is more subjective and should be guided by the extent of disease at the prostate base and whether the seminal vesicles are involved (56).

In accordance with the well-described dose-escalation trials for primary RT of localized prostate cancer, it has recently been proposed that dose intensification either for SRT or ART would be more effective in terms of cancer control (57).

Also, it has been suggested that each Gy increase in total dose may improve the BPFS by more than 3% (58). Therefore, a total dose toward 70 Gy might be considered in the salvage situation, when the risk of severe toxicity can be minimized by using modern radiation techniques. In the absence of results from randomized trials, the potentially improved local tumor control by a higher RT dose should be carefully weighted up against possibly increased toxicity.

An increase in the RT dose will certainly increase grade 3 or more late toxicity. In a retrospective study where 70 Gy were administered using intensity-modulated radiation therapy (IMRT) to the prostate bed, urinary incontinence reached 13% and erectile dysfunction 26% (59). With higher doses of IMRT 76 Gy, the genitourinary and intestinal toxicity increased to 22 and 8% of the patients, respectively (60).

Although theoretical assumptions might claim a benefit in escalating the RT dose, a randomized trial is needed to definitely answer this question. The Swiss Group for Clinical Cancer Research (SAKK) conducted a randomized controlled international trial comparing SRT with 64 vs. 70 Gy without ADT in patients with prostate cancer and biochemical relapse after RP (SAKK 09/10, NCT01272050). The trial included men ≤75 years with pT2–3 N0 R0-1, with a PSA of at least ≥0.1 ng/ml and above but not higher than 2 ng/ml. Patients with evidence of macroscopic recurrence or metastatic disease were excluded. The primary endpoint was freedom from biochemical progression including a PSA of ≥0.4 ng/ml and above and/or clinical failure. The trial included quality of life analysis, quality assurance of RT, and a central pathology review. Three-dimensional conformal or IMRT were allowed per protocol. Three hundred and forty-four patients were randomized. The 13% grade 2 acute genitourinary toxicity and 0.6% grade 3 acute intestinal toxicity with 64 Gy were reported in comparison to 16.6% grade 2 and 1.7% grade 3 genitourinary toxicity with 70 Gy (*p* = 0.2). The 16% grade 2 acute intestinal toxicity and 0.6% grade 3 acute intestinal toxicity with 64 Gy were reported in comparison to 15.4% grade 2 and 2.3% grade 3 with 70 Gy (*p* = 0.8). Patients who received 70 Gy reported a more pronounced and clinically relevant genitourinary toxicity (mean difference in change score between arms, 3.6; *p* = 0.02) (61). Considering that this is an early report on toxicity, long-term toxicity as well as efficacy analysis is still pending.

## Current Phase III Studies for PSA Relapse

The Radiation Therapy Oncology Group completed a phase III clinical trial (RTOG 9601) comparing ART with SRT (64.6 Gy in 36 fractions) plus 2 years of a high dose bicalutamide (150 mg per day) for patients with post-RP PSA relapse. The study group included patients with PSA levels from 0.2 to 4.0 ng/ml with prostate tumors classified as either pT2pN0 and a positive surgical margin or pT3pN0. The study closed in 2003 after accruing a total of 840 patients. Its final publication is pending at present. A recent presentation at the American Society for Radiation Oncology (ASTRO) reported that at a median follow-up of 12.6 years, there was an improvement in OS of 82% for the RT plus ADT vs. 78% for the RT plus placebo patients; with a hazard ratio of 0.75 (95 percent CI: 0.58–0.98) with a two-sided *p*-value = 0.036. Data indicated that the addition of ADT decreased the rate of death by prostate cancer and decreased the risk of the cancer metastasizing. The 12-year incidence of prostate cancer centrally-reviewed deaths was 2.3% for the RT plus ADT group, compared to 7.5% for the radiation plus placebo group (*p* < 0.001). At 12 years, the cancer had metastasized in 51 patients (14%) in the RT plus ADT group, compared to 83 patients (23%) in the radiation plus placebo group (*p* < 0.001). Additionally, late grade 3 and grade 4 bladder and bowel side effects were similar in both groups, whereas 70% of men in the RT plus ADT reported swelling of the breasts, compared to 11% from the radiation plus placebo group (62).

Currently, the RTOG is conducting another phase III, three-arm, study (RTOG 0534) to examine the potential benefit of adding 4–6 months of ADT to SRT and to address a potential role of treating pelvic lymph nodes. The United Kingdom is conducting a phase III study called RADICALS (Radiotherapy and Androgen Deprivation in Combination After Local Surgery), and part of this study is to assess the benefit of adding 6-months or 24-months of ADT to SRT. A French group is conducting a phase III study comparing SRT with SRT plus 6-months of ADT (Clinical Trials Gov. Identifier: NCT00423475). Unfortunately, the EORTC 22043, a two-arm phase III trial, which compared 6 months of ADT concomitant to ART, closed in 2014 due to lack of patient accrual.

## Conclusion

Radiotherapy represents a curative approach to treat prostate cancer in patients with postoperative detectable PSA. However, its efficacy is affected by the presence of adverse clinical/pathological prognostic factors. In this context, a patient with PSA relapse after RP represents a clinical dilemma. Treatment decisions have been jeopardized by a variety of retrospective trials that have used different postoperative PSA cut-off points and the lack of clear evidence demonstrating which therapeutic attitude is best, particularly in prolonging the patient’s life without significant side effects. The use of ART over observation has been proven to prolong BPFS in phase III randomized trials, but its benefit in prolonging OS has also been questioned. The challenge of managing these patients in current clinical practice will be solved in the near future when the results of different on-going randomized trials become available.

## Author Contributions

FH and DB both collected the data and drafted the manuscript. DB approved the submitted version of the manuscript.

## Conflict of Interest Statement

The authors declare that the research was conducted in the absence of any commercial or financial relationships that could be construed as a potential conflict of interest.
